# The design and protocol of heat-sensitive moxibustion for knee osteoarthritis: a multicenter randomized controlled trial on the rules of selecting moxibustion location

**DOI:** 10.1186/1472-6882-10-32

**Published:** 2010-06-25

**Authors:** Rixin Chen, Mingren Chen, Mingfei Kang, Jun Xiong, Zhenhai Chi, Bo Zhang, Yong Fu

**Affiliations:** 1 Affiliated Hospital with Jiangxi University of TCM, Nanchang, PR China

## Abstract

**Background:**

Knee osteoarthritis is a major cause of pain and functional limitation. Complementary and alternative medical approaches have been employed to relieve symptoms and to avoid the side effects of conventional medication. Moxibustion has been widely used to treat patients with knee osteoarthritis. Our past researches suggested heat-sensitive moxibustion might be superior to the conventional moxibustion. Our objective is to investigate the effectiveness of heat-sensitive moxibustion compared with conventional moxibustion or conventional drug treatment.

**Methods:**

This study consists of a multi-centre (four centers in China), randomised, controlled trial with three parallel arms (A: heat-sensitive moxibustion; B: conventional moxibustion; C: conventional drug group). The moxibustion locations are different from A and B. Group A selects heat-sensitization acupoint from the region consisting of Yin Lingquan(SP9), Yang Lingquan(GB34), Liang Qiu(ST34), and Xue Hai (SP10). Meanwhile, fixed acupoints are used in group B, that is Xi Yan (EX-LE5) and He Ding (EX-LE2). The conventional drug group treats with intra-articular Sodium Hyaluronate injection. The outcome measures above will be assessed before the treatment, the 30 days of the last moxibustion session and 6 months after the last moxibustion session.

**Discussion:**

This trial will utilize high quality trial methodologies in accordance with CONSORT guidelines. It will provide evidence for the effectiveness of moxibustion as a treatment for moderate and severe knee osteoarthritis. Moreover, the result will clarify the rules of heat-sensitive moxibustion location to improve the therapeutic effect with suspended moxibustion, and propose a new concept and a new theory of moxibustion to guide clinical practices.

**Trial Registration:**

The trial is registered at Controlled Clinical Trials: ChiCTR-TRC-00000600.

## Background

Osteoarthritis (OA) is the most common form of arthritis [1] and the leading cause of disability among older adults [2,3]. As one part of weight-bearing peripheral and axial joints, knee is the most commonly affected by osteoarthritis [4]. Among adults aged 30 years, symptomatic knee OA occurs in 6% and symptomatic hip OA in about 3%[4].Knee osteoarthritis (KOA) is associated with symptoms of pain and functional disability. Physical disability arising from pain and loss of functional capacity reduces the quality of life and increases the risk of further morbidity and mortality [5]. The prevalence, disability, and associated costs of KOA are expected to steadily increase over the next 25 years because of aging in the population [6]. After adjusting for age, sex, and comorbidity, KOA is responsible for a higher percentage of disability than any other medical condition for the following activities: stair climbing, walking a mile, and housekeeping.

The underlying disease processes of KOA involve cartilage degeneration, proliferation and remodeling of subchondral bone structure. Recently there is no cure for KOA [7]. Therefore, the treatment of KOA is primarily focused on managing the condition by minimizing morbidity. The current conventional treatment of KOA symptoms and analgesics, such as NSAIDS, glucosamine, topical analgesics, intra-articular (Sodium Hyaluronate, Synvisc) and surgical treatment[8,9]. Substantial numbers of patients with KOA are not satisfied with conventional drug treatment and repeatedly experience side effects [10,11]. As a result, a large number of patients with KOA are turning to complementary and alternative treatments. Non-pharmacological treatments such as acupuncture are therefore attractive. Acupuncture is often used for KOA. For example, it is gaining popularity among KOA patients in the US and about 1 million consumers utilize acupuncture annually which has musculoskeletal disorders [12].

Acupuncture is a safe treatment that has a low risk for serious side effects. Moxibustion is a traditional Chinese method of acupuncture treatment, which utilizes the heat generated by burning Moxa (it is also called Mugwort or Moxa) to stimulate the acupuncture points. The technique consists of lighting a moxa stick and bringing it close to the skin until it produces hyperaemia due to local vasodilatation. The intensity of moxibustion is just below the individual tolerability threshold. Moxibustion has anti-inflammatory or immunomodulatory effects against chronic inflammatory conditions in humans [13]. Moreover, the heat of moxa treatment improves microcirculation in the knee. Therefore, these Arthritis substances may be reduced and weakened by moxibustion. Then Elimination of swelling and pain relief also may be achieved. Especially for swell type KOA, which derived from surrounding tissues strain, moxibustion may get a better effect. Further deterioration of cartilage is set back, as a result of pathological chain of KOA is cut in treatment of moxibustion. That is to say, moxibustion does not make osteophyte disappeared in short treatment, but its therapeutic effects relieve the main symptoms, and minimizing morbidity of new osteophyte by avoiding pathological product of stimulus and mechanical structural imbalance.

Although firm evidence has not been established, the results of some systematic review and meta-analysis suggest that acupuncture and moxibustion may effective in the treatment of KOA [14-16].Such as the latest meta-analysis concluded that sham-controlled trials showed clinically irrelevant short-term benefits of acupuncture for treating knee osteoarthritis. Waiting list controlled trials suggested clinically relevant benefits, some of which may be due to placebo or expectation effects [16]. However, these reviews do not confirm the efficacy of acupuncture and moxibustion. This may be because all relevant RCTs were limited by methodological defects, including inappropriate sample size, variability of acupuncture and sham protocols, and missing information. Therefore, rigorous high-quality randomised controlled trials are needed.

Thinking about moxibustion itself, the selection of location for manipulating Moxa plays an important role in obtaining good effects [17]. In our opinion, the nature of acupoint is not location , but status. In the human being there are two functional states, sensitization state and rest state. When the human body has disease, acupoints on the body surface may be sensitized with various types of sensitization, and acupoint heat-sensitization is a type of acupoint sensitization. The sensitized acupoints show acupoint-specific "small stimulation inducing large response" for external relative stimulation. The Inner Canon of Huangdi or Yellow Emperor's Inner Canon is an ancient Chinese medical text that has been treated as the fundamental doctrinal source for Chinese medicine for more than two millennia and until today. According with its core viewpoint and theory, acupoint is described and understood with the state , which is certain area of the skin in the course of diseases. Among the changes, sensitized status is the common one, described that acupoints on the body surface may be sensitized with various types of sensitization. This sensitized acupoint is not only the pathological phenomenon reflecting the diseases but also stimulating location with acupuncture and moxibustion. Acupoint heat-sensitization is a type of acupoint sensitization.

Our research term found that the heat-sensitized phenomenon was a new type of acupoint sensitized features in pathological state [18-20]. We applicated the acupoint heat-sensitization phenomenon and rule in the past twenty years. The past experiential evidences in our research indicated that the functional state might jump from the rest state to the heat-sensitized state suffering from diseases. Its characteristic was thought that these special acupoints might produce heat response and farther warm sensation, as a result of stimulation of moxibustion heat. If we can search out these heat-sensitized acupoints associating with pathological state, good effect will be achieved. Therefore, selecting the heat-sensitized acupoint may obtain therapeutic effect far better than acupuncture and moxibustion at acupoints of routine rest state. We carried our many clinical trials to test and verify the efficacy heat-sensitized acupoint, such as myofascial pain syndrome [21], lumbar disc herniation[22], pressure sores[23] and KOA[24]. The result of clinical trials almost suggested superiority effect of heat-sensitized acupoint and encouraged us to proceed.

With these constraints, we planned a rigorous multi-centre randomised controlled trial with a large sample size.

## Method/design

### Objective

The aim of this study is to investigate the effectiveness of heat-sensitive moxibustion compared with conventional moxibustion or conventional drug treatment (intra-articular Sodium Hyaluronate injection) in patients with moderate to severe KOA in China.

### Outcome

Ministry of Health of the People's Republic of China (MHPRC) has proposed a series of criteria to define patient response in the context of clinical trials of KOA, known as the guiding principle of clinical research on new drugs (GPCRND) [25]. According to these criteria, a patient with KOA is assessed including pain, the relation between activity and pain, function impairment, and special exams (Table 1). This scoring system was previously validated. The degree of KOA is divided into three level: mild- < 5 score;moderate-5 ~ 9 score; severe-> 9 score.

**Table 1:** List of GPCRND-KOA

Item	Grade/Classification	Score
Pain or discomfort in night when lying in bed	No	0
	Pain in activity or some position	1
	Pain in non-activity	2
Morning stiffness or pain worse when getting out of bed	No	0
	< 30 minutes	1
	≥ 30 minutes	2
Pain or discomfort in walk	No	0
	After walking in some distance	1
	Pain at beginning of walk or worse	2
Arise from seat	Independent	0
	Need assistance	1
The maximum walk distance(accompany with pain)	Unrestricted	0
	Restricted, > 1 km	1
	300 m ~ 1 km	2
	< 300 m	3
Daily Activities	Board standard airstairs	
	Independent	0
	Difficulty	1
	Unable	2
	Step down standard airstairs	
	Independent	0
	Difficulty	1
	Unable	2
	Squat or bend knees	
	Independent	0
	Difficulty	1
	Unable	2
	Walk over rough terrain	
	Independent	0
	Difficulty	1
	Unable	2

Therapeutic effect was assessed by comparing baseline and final conditions reported by the patient. Four categories were listed as below: clinical response-no symptom, normal function activity, clinical symptom score reduction ≥ 95%; markedly effective-no obvious symptom, normal joint function activity, able to participate in activity and work, clinical symptom score reduction 70 ~ 95%; improved-no pain, normal joints' flexion and extension, improvement in activity and work, clinical symptom score reduction 30 ~ 70%; ineffective-not arrived at the above standard involving with symptom and function impairment, clinical symptom score reduction < 30%.

This trial also records adverse effects reported by patients during treatment. The outcome measures above will be assessed before the treatment, the 30 days of the last moxibustion session and 6 months after the last moxibustion session.

### Design

**Figure 1:**
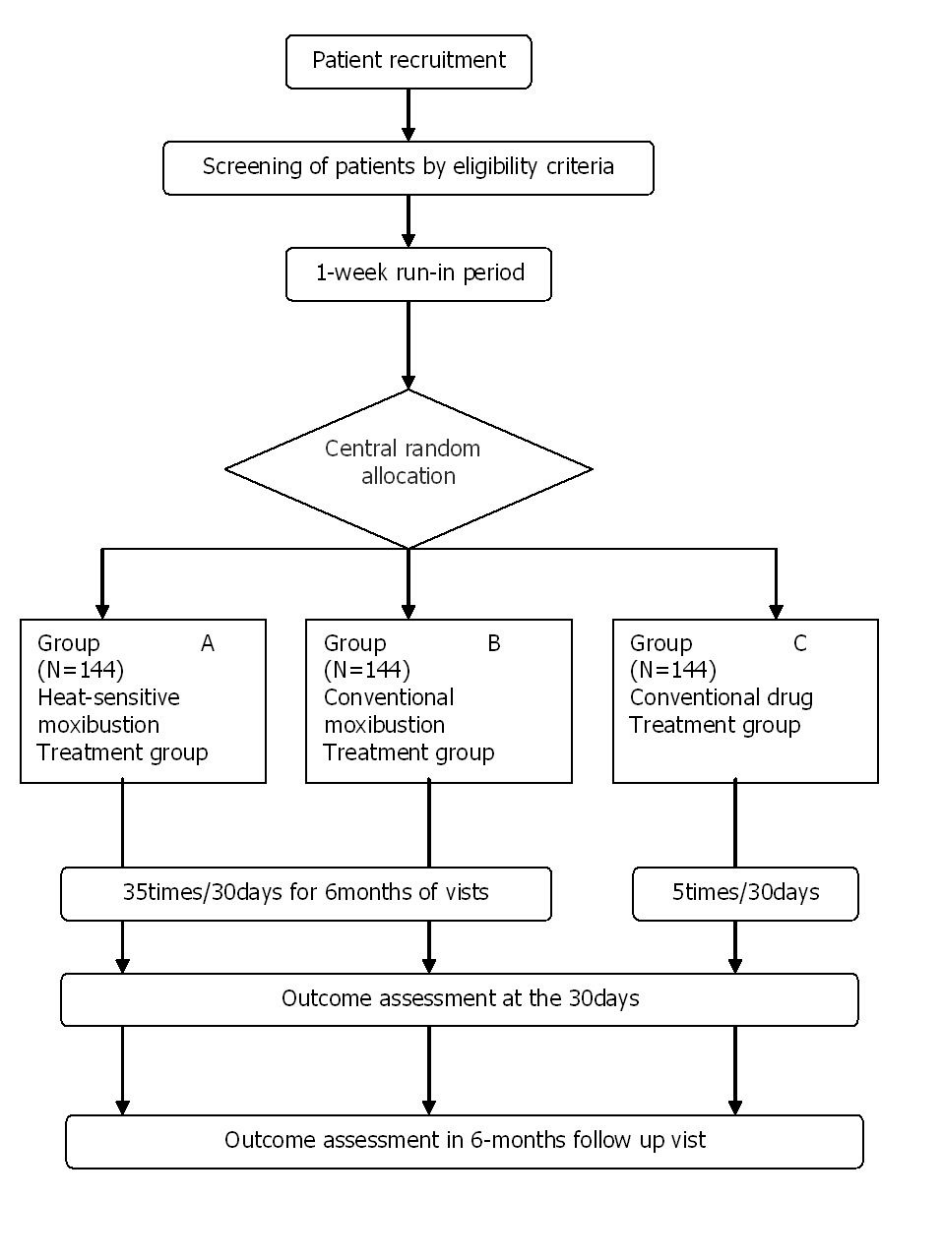
**The flow diagram is intended to depict the passage of participants through this RCT**.

A multi-centre (four centers in China), randomised, subject blinded(group A and B) and assessor blinded, positive controlled trial will be conducted at the Jiangxi Traditional Chinese Medicine Hospital in Nanchang, The first Affiliated Hospital with Anhui University of TCM in Hefei, Jiangsu Traditional Chinese Medicine Hospital in Nanjing, and Shanxi Traditional Chinese Medicine Hospital in Xian. The study will be sequentially conducted as follows: a run-in period of one week prior to randomisation, a treatment period of 30 days (5 sessions per week), and a follow-up period of six months. The total study period will be eight months. At the end of the run-in period, participants will be randomised to the heat-sensitive moxibustion group, the conventional moxibustion group, or the conventional drug treatment group by the central randomization system (Figure 1).This system is provided by China Academy of Chinese Medical Sciences, which adopted the computer telephone integration (CTI) technology to integrate computer, internet and telecom. The random number list will be assign by interactive voice response (IVR) and interactive web response (IWR)[26]. The success of blinding will be assessed at each participant's last visit. Researchers who did not participate in the treatment and who are blinded to the allocation results will perform the outcome assessment

### Eligibility

#### Inclusion criteria

Eligible participants will be those previously diagnosed with moderate to severe KOA, according to the GPCRND-KOA criteria(> 5 score). Patients will be required to complete the baseline KOA diary. Written informed consent will be obtained from each participant. , Participants 38 ~ 70 years of age will be recruited from outpatient and inpatient in four centers. Standard of diagnosis listed as follows:(1) Keen pain almost occurred in the past one month; (2)Osteophyma at the edge of joints are all well demonstrated in X-ray; (3)Laboratory examinations of arthritis support the diagnosis of osteoarthritis;(4) Morning stiffness continued less than 30 minutes;(5) Bone sound existed when joints was taking flexion and/or extension. If a patient accord with (1), (2), or (1), (3), (4), (5), we can diagnose. Since the research involves moderate to severe KOA or severe KOA, the inclusion criteria will restrict the following conditions: According with the below KOA diagnosis standard, meanwhile, knee joints appear die Schwellung; Floating patella test is negative; Patients accept the treatment protocol in this trial; Acupoint heat-sensitization phenomenon exists in the region consisting of Yin Lingquan(SP9), Yang Lingquan(GB34), Liang Qiu(ST34), and Xue Hai (SP10).

Participants will be instructed to stop KOA symptomatic relief medication during the run-in and treatment periods and will be provided the usual care instruction for KOA.

#### Exclusion criteria

Participants will be excluded if they suffer from serious life-threatening disease, such as disease of the heart and brain blood vessels, liver, kidney and hematopoietic system, and psychotic patients. Participants will not be eligible if the female are in the duration of pregnancy or lactation. The following conditions are also excluded items: acute knee joint trauma or ulceration in its local skin; complicated with serious genu varus/valgue and flexion conttration.

### Treatment protocol

#### Heat-sensitive moxibustion

Moxibustion will be performed by certified acupuncture medical doctors at four centers. Qualified specialists of acupuncture in traditional Chinese medicine with at least five years of clinical experience will perform the acupuncture in this study. All treatment regimens will be standardized between four centers practitioners via video, hands-on training and internet workshops. Participants will be randomly assigned to the heat-sensitive moxibustion group, the conventional moxibustion group, or the conventional drug group. In the former groups, a 22 mm (diameter) × 160mm (length) moxa-sticks (Jiangxi Traditional Chinese Medicine Hospital, China) will be used. The patient is usually in the comfortable supine position for treatment, with 24°C ~ 30°C temperature in the room. He should be wearing loose trousers, especially making his knee joints exposed.

For the heat-sensitive moxibustion group, the moxa-sticks are lit by the therapist and held over the region consisting of Yin Lingquan(SP9), Yang Lingquan(GB34), Liang Qiu(ST34), and Xue Hai (SP10). The warming suspended moxibustion about distance of 3cm are used to search the acupoint heat-sensitization phenomenon. The following patients sensation will suggest the special heat-sensitization aupoint: diathermanous sensation due to moxa-heat, defining as the heat sensation conducting from the moxa local skin surface into deep tissue, or even into the joint cavity; expand heat sensation due to moxa-heat, defining as the heat sensation spreading the surrounding little by little around the moxa point; transfer heat sensation due to moxa-heat, defining as the heat sensation transferring along some pathway or direction, even to the ankle or hip conduction; non-heat sensation due to moxa-heat. When some acupoint exists one below sensation at least, the therapists mark the point as heat-sensitive acupoint. We try our best to seek all the special acupoints in each patient by the repeated manipulation.

The therapists begin to treat patients from the most heat-sensitive intensity acupoint. Treatment sessions end when patients are feel the acupoint heat-sensitization phenomenon disappeared. Generally speaking, we find the time range from 30 ~ 60 minutes.Patients receive the treatment two times/day in 1^st ^week(two times/day from 2^nd ^week ) for a total of 35 sessions over 30 days.

#### Conventional moxibustion group

Patients assigning in conventional moxibustion group will receive fixed acupoint moxibustion. Common practices are similar with the first group. The different manipulation is that the therapists carry out warming moxibustion in traditional acupoint, selecting Xi Yan (EX-LE5) and He Ding (EX-LE2). One point is treated 15minutes a time. Three points are treated by suspended moxibustion at the same time. In the treatment, the therapists make patients the same intensity of local warm sensation as the former group. The sensation of acupoint heat-sensitization phenomenon is not pursued and not avoided in the treatment. Patients receive the treatment two times/day in 1^st ^week (two times/day from 2^nd ^week) for a total of 35 sessions over 30 days.

#### Conventional drug group

Now, intra-articular hyaluronan (HA) or hylan is popular approved for the treatment of KOA [27]. A number of systematic reviews have published positive evidence of efficacy and safety of intra-articular HA for KOA. For example, a recent review proved HA to be an effective, safe, and tolerable treatment for symptomatic KOA [28]. Therefore, this protocol selected Sodium Hyaluronate Injection Intra-articula as the conventional drug. The injection will be used six days a time (2ml) as a total of five times.

### Statistical methods

#### Analysis

We will conduct analysis on an intention-to-treat basis (significance level p < 0.05) using the SAS statistical package program (ver. 9.1.3).The analysis of center effect will be used Cochran-Mantel-Haenszel. Baseline characteristics will be shown as mean ± standard deviation (SD) for continuous data including age, previous duration, and GPCRND-KOA criteria. As for participants' gender, n (%) of male and female in each group will be shown as baseline characteristics. We will conduct between-group comparision in baseline using ANCOVA (analysis of covariance) for continuous data and using Chi-square test or nonparametric test for gender composition considering p < 0.05 as statistically significant.

For outcome measures, the mean differences from baseline values to the end of treatment will be compared using ANCOVA. If any imbalances in baseline characteristics between groups are encountered, we will conduct ANCOVA (analysis of covariance) using these imbalanced variables as covariates and allocated group as fixed factor.

#### Data integrity

The integrity of trial data will be monitored by regularly scrutinizing data sheets for omissions and errors. Data will be double-entered and the source of any inconsistencies will be explored and resolved.

#### Sample size

We wished to estimate the sample size that would suffice to detect GPCRND-KOA between the heat-sensitive and conventional moxibustion groups. In our previous pilot study, the effective rate in heat-sensitive moxibustion group is 70%, and 50% in the other group. If we apply a two-sided 5% significance level, 95% power the calculated required sample size is approximately 120 participants in each group, according to the following equation. Allowing for a 20% loss to follow up, a total of 144 participants will be required in each group, with 432 participants in total.

### Adverse events

We define adverse events as unfavorable or unintended signs, symptoms or disease occurring after treatment that are not necessarily related to the moxibustion intervention. In every visit, adverse events will be reported by participants and examined by the practitioner.

### Ethics

Written consent will be obtained from each participant. This study was approved by all relevant local ethics review boards. Ethics Committee of Affiliated hospital of Jiangxi Institute of Traditional Chinese Medicine had approved this trial: code issued by ethic committee is 2008(9).

## Discussions

To our knowledge, although there is no cure for KOA, current kinds of therapies focus on the relief of pain and stiffness and maintenance or improvement in functional status and quality of life as important goals. A number of clinical trial suggested moxibustion as one of traditional acupuncture therapy, should effective in the treatment of KOA. But the methodological problems of published trials haunt us the trust of moxibustion. Therefore, we design this rigorous clinical trials meeting the CONSORT statement and guidelines to guarantee a high internal validity for the results. In view of the special nature of the KOA itself, positive control should be selected to solve the patient's pain, swell, and impairment. At present, various conventional medications are used to slow down and control with the disease. Although Anesthetic and NSAIDS can relive the pain and inflammation, patients are not considered it as priority options because of multiple adverse reactions. Sodium Hyaluronate acid injections may give you more pain relief than oral medicines, and less adverse reactions. Therefore, we used the Sodium Hyaluronate acid injections as drug treatment in the protocol.

Actually, the aim of this trial contains two parts: "Is moxibustion treatment superior to Sodium Hyaluronate acid injections for KOA? And is heat-sensitive moxibustion superior to conventional moxibustion for KOA?" The latter one is our more interesting goal. According to the current theory of traditional Chinese medicine, moxibustion resulting from the burning of moxa, produces the radiant heat and drug effects to acupoints. This treatment penetrates deeply into the body, restoring the balance and flow of vital energy or life force through acupoints. So the selected of location for manipulating Moxa plays an important role in obtaining good effects. Generally speaking, the location acupoints are fixed along meridians. The conventional moxibustion is promoted for improving general health and treating diseases by stimulating these fixed acupoints. And doctors consider hyperaemia due to local skin vasodilatation as the indicator of moxibustion's effect. However, our clinical experience and observation in the past suggested that stimulating these fixed acupoints might not the best treatment site for moxibustion. In the human being there are two functional states, sensitization state and rest state. When the human body has disease, acupoints on the body surface may be sensitized with various types of sensitization, and acupoint heat-sensitization is a type of acupoint sensitization. acupoint is more than fixed skin site but external sensitive point reflecting the diseases. Therefore, acupoint is variable and depends on the pathological state. Traditional fixed acupints are thought as indicators to searching specific sensitive acupoint. That is, traditional fixed acupints don not consider the state as the key factor to local the acupoint, so the course of fixing the position is imprecisely.

When we light the moxa hold over the heat-sensitive acupoints, the patients will produce some heat-sensitization phenomenon. The following patients sensation will suggest the special heat-sensitization aupoint: diathermanous sensation due to moxa-heat, defining as the heat sensation conducting from the moxa local skin surface into deep tissue, or even into the joint cavity; expand heat sensation due to moxa-heat, defining as the heat sensation spreading the surrounding little by little around the moxa point; transfer heat sensation due to moxa-heat, defining as the heat sensation transferring along some pathway or direction, even to the ankle or hip conduction; non-heat sensation due to moxa-heat.

It is well-known that acupuncture and moxibustion originated from China several thousands years ago. The Inner Canon of Huangdi or Yellow Emperor's Inner Canon is an ancient Chinese medical text that has been treated as the fundamental doctrinal source for Chinese medicine for more than two millennia and until today. According with its core viewpoint and theory, acupoint is described and understood with the state , which is certain area of the skin in the course of diseases. "*Said section (acupoint) who shen-qi out of the procession itself, not flesh bones *"(Huang Di Neijing, Lingshu, Chapter: jiu zhen shi er yuan).That is, acupoints are not flesh bones, which have their particular morphology and fixed location, but dynamic functional state due to shen-qi's activity. "*when five internal organs are suffering from diseases, we can use 12 yuan-acupoints to treat. These acupoints are derived from five internal organs intrinsic nature. Five internal organs are diagnosed by 12 yuan-acupoints. Different organs accord with relative yuan-acupoint. We can observe and distinguish the acupoint response to external stimulation. Then, the disease from five internal organs can be diagnosed and treated by 12 yuan-acupoints" *(Huang Di Neijing, Lingshu, Chapter: jiu zhen shi er yuan).So an important view was expressed by this sentence, that is, acupoints reflect the internal diseases and can be operated in treatment of disease with the functional role. In physiological status, people do not always become aware of the existence of acupoint. In contrast, patients can feel some changes from acupoint involving with diseases in pathological state. So the ancient persons located the acupoints through the state changes. "*Lung shu-acupoints locate nearby the third vertebrae; Heart shu-acupoints locate nearby the fifth vertebrae; Ge shu-acupoints locate nearby the seventh; Liver shu-acupoints locate nearby the ninth vertebrae; Spleen shu-acupoints locate nearby the eleventh vertebrae; Kindey shu-acupoints locate nearby the fourteenth vertebrae. These acupoints are far from about three-inch by spinal crest. If you want to search our acupoints exactly, the finger's touch and press are must be use above the skin and we obtain the sensation from patients, such as pain. " *(Huang Di Neijing, Lingshu, Chapter: back shu). Back shu acupoints are searched by doctor through external stimulation. "If a patient is coughing with shoulder, we can select the lateral chest shu acupoints to treat. The shu acupoints are nearby from *the third vertebrae to the fifth vertebrae. Press the location, patients will be comfortable at once. Then we needle the location, which is acupoint actually*." (Huang Di Neijing, Lingshu, Chapter: five xie) *We conclude that *sensitized status is key factor to locate the position of acupoints.

Among the changes, sensitized status is the common one, described that acupoints on the body surface may be sensitized with various types of sensitization. This sensitized acupoint is not only the pathological phenomenon reflecting the diseases but also stimulating location with acupuncture and moxibustion. Acupoint heat-sensitization is a type of acupoint sensitization, which derived from our clinical experience and research in past twenty years. The special acupoint makes accordance with the classical thought and theory from the Inner Canon of Huangdi.

Our empirical evidence engaged us to formulate the following hypothesis: selecting the heat-sensitized acupoint may obtain therapeutic effect far better than acupuncture and moxibustion at acupoints of routine rest state. The main aim of this trial is to test and verify the hypothesis. If we can confirm this hypothesis, promoting moxibustion clinical efficacy would be carried our in this extraordinary way. It is of great significance to develop the theory of acupuncture and moxibustion.

Therefore, the purpose of this trial is more than discuss the efficacy of moxibustion as treatment. The results of our trial will be helpful to supply the evidence on the rules of heat-sensitive moxibustion location in China.

## Competing interests

The authors declare that they have no competing interests.

## Authors' contributions

RC and MC obtained funding for the research project and drafted the protocol. JX wrote the final manuscript. MK contributed to the research design and made critical revisions. ZC, BZ and YF were responsible for the statistical design of the trial and wrote portions of the statistical methods, data handling, and monitoring sections. All authors read and approved the final manuscript.

## Pre-publication history

The pre-publication history for this paper can be accessed here:

http://www.biomedcentral.com/1472-6882/10/32/prepub
